# Vaginal Mucosal Homeostatic Response May Determine Pregnancy Outcome in Women With Bacterial Vaginosis: A Pilot Study

**DOI:** 10.1097/MD.0000000000002668

**Published:** 2016-02-08

**Authors:** Emmanuel Faure, Karine Faure, Martin Figeac, Eric Kipnis, Teddy Grandjean, Sylvain Dubucquoi, Céline Villenet, Bruno Grandbastien, Gilles Brabant, Damien Subtil, Rodrigue Dessein

**Affiliations:** From the Faculty of Medicine of Lille, EA7366 Host-Pathogen Translational Research Group, University Lille North of France (EF, KF, EK, TG, RD); CHRU de Lille, Service de Maladies Infectieuses, Hôpital Claude Huriez (EF, KF, RD); University Lille, CHU Lille, IRCL, Structural and Functional Genomics Core Facility (MF, CV, RD); CHU Lille, Institut d’Immunologie—Centre de Biologie Pathologie et Génétique (SD, RD); UDSL, EA 2686, UFR Médecine (SD, RD); Univ Lille Nord de France (SD, BG, RD, DS); CHU Lille, Institut de Microbiologie, Laboratoire de Bactériologie Hygiène, Centre de Biologie Pathologie et Génétique (RD); UDSL, UFR Médecine (RD); CHU Lille, Service de Gestion du Risque Infectieux, des Vigilances et d’Infectiologie (BG); UDSL, EA 2694, UFR Médecine (BG, DS); Hôpital Saint Vincent, Service de Gynécologie-Obstétrique (GB); CHU Lille, Service de Gynécologie-Obstétrique Hôpital Jeanne de Flandre (DS), Lille, France.

## Abstract

Bacterial vaginosis (BV) is considered as a trigger for an inflammatory response that could promote adverse pregnancy outcome (APO). We hypothesized that BV-related inflammation could be counterbalanced by anti-inflammatory and mucosal homeostatic responses that could participate in pregnancy outcomes.

A total of 402 vaginal self-samples from pregnant women in their first trimester were screened by Nugent score. In this population, we enrolled 23 pregnant women with BV but without APO, 5 pregnant women with BV and developing APO, 21 pregnant women with intermediate flora, and 28 random control samples from pregnant women without BV or APO.

BV without APO in pregnant women was associated with 28-fold interleukin-8, 5-fold interleukin-10, and 40-fold interleukin-22 increases in expression compared to controls. BV associated with APO in pregnant women shared 4-fold increase in tumor necrosis factor, 100-fold decrease in interleukin-10, and no variation in interleukin-22 expressions compared to controls. Next-generation sequencing of vaginal microbiota revealed a shift from obligate anaerobic bacteria dominance in BV without APO pregnant women to *Lactobacillus* dominance microbiota in BV with APO.

Our results show that the anti-inflammatory and mucosal homeostatic responses to BV may determine outcome of pregnancy in the setting of BV possibly through effects on the vaginal microbiota.

## INTRODUCTION

Bacterial vaginosis (BV) is a dysbiosis of the vaginal microbiota characterized by a shift from the normal *Lactobacillus*-dominant species to an increased abundance of anaerobic species and genital mycoplasmas.^1^ BV is a risk factor for *Trichomonas vaginalis*, *Neisseria gonorrhoeae*, *Chlamydia trachomatis*, human papillomavirus, herpes simplex virus type-2, human immunodeficiency virus infection, pelvic inflammatory disease, and posthysterectomy wound infections in women of reproductive age.^2^ During pregnancy, while most BV remain clinically asymptomatic, the occurrence of BV before 13 completed weeks of amenorrhea increases the risk of adverse pregnancy outcomes (APOs) including preterm birth, spontaneous preterm labor, preterm prelabor rupture of membranes, recurrent abortion, or miscarriage.^3^ However, the pathophysiological mechanism through which BV affects the ongoing pregnancy remains unclear. To date, BV has only been considered as a trigger for an adverse inflammatory response during pregnancy. Indeed, an excess of proinflammatory cytokines during pregnancy is linked to APO.^4,5^ However, during pregnancy the immune balance is in favor of an anti-inflammatory/immunosuppressive state to tolerate foreign paternal antigens present in the fetus.^6^ Therefore, the interaction between the dysbiosis of BV and the specific anti-inflammatory balance of pregnancy might be involved. In other settings of mucosal microbiotal dysbiosis/immune interaction such as inflammatory bowel diseases, the critical immune responses involve proinflammatory cytokines, interleukin-17 (IL-17), interleukin-18 (IL-18), interleukin-6 (IL-6), interleukin-8 (IL-8), interleukin-1β (IL-1β), tumor necrosis factor (TNF); mucosal wound repair cytokines such as interleukin-22 (IL-22); and anti-inflammatory cytokines such as interleukin-10 (IL-10).^7,8^ Therefore, in a pilot study of pregnant women with BV in their first trimester, we studied the relation between the vaginal mucosal immune balance and pregnancy outcome.

## MATERIALS AND METHODS

### Patient Selection

We screened BV in all pregnant women ages 18 years or older with a gestational age of <13 completed weeks of amenorrhea at the time of screening during routine pregnancy follow-up consults. During the consult, a short-standardized questionnaire collected the age, the educational level, whether they had smoked since the beginning of pregnancy, and whether they had any history of APO. APO including preterm birth, spontaneous preterm labor, preterm premature rupture of membranes, recurrent abortion or miscarriage, and antibiotic therapy before the delivery were collected 10 months later from the medical records. Each woman performed a vaginal self-sample with 2 swabs. One swab was used to identify BV, intermediate flora (IF), and normal flora using the Nugent score. Following the constitution of the groups of women with BV and IF, a control group of an equal number samples was randomly sampled from the much larger pool of swabs with normal Nugent score. The second vaginal swab was discharged in 1 mL of transport media (Eswab, Coban, Italy) and stored at −80°C for ribonucleic acid (RNA) extraction and flow cytometry.

### Nugent Score

Vaginal secretions were spread on a clean microscopy slide and heat-fixed within 4 h. The vaginal smear was then Gram-stained. Examining several microscopic fields at a 10-fold magnification assessed bacterial and cellular abundance. Nugent score was established as described elsewhere.^9^ Scores of 0 to 3 were considered normal (lactobacillus dominant), 4 to 6 were labeled as intermediate (mixed morphotypes), and 7 to 10 were indicative of BV (absence of lactobacilli and predominance of the other 2 morphotypes).

### Quantitative Real-Time Polymerase Chain Reaction

RNA extractions were performed using GeneJet RNA Purification Kit (Fermentas, Thermo fisher scientific Waltham, MA, USA, K0731). Isolated RNAs were reverse-transcribed to complementary deoxyribonucleic acid (cDNA) with the High-Capacity cDNA Archive kit (Applied Biosystems, Foster City, CA). The resulting cDNA (equivalent to 5 ng of total RNA) was amplified using the SYBR Green Real-Time Polymerase Chain Reaction kit and detected on a Stratagene Mx3005P (Agilent Technologies, Santa Clara, CA). RNA quality was assessed using a NanoDrop ND-8000 spectrophotometer. Real-time polymerase chain reaction was performed with the forward and reverse primers that were designed using Primer express software, version 1.0 (Applied Biosystems).

Specific primers for: glyceraldehyde 3-phosphate dehydrogenase (GAPDH) (5′-ACCCACTCCTCCACCTTTGA-3′ and 3′-CATACCAGGAAATGAGCTTGACAA-5′), TNF (5′-CCCAGGCAGTCAGATCATCTTC-3′ and 5′-AGCTGCCCCTCAGCTTGA-3′), IL-17 (5′-ACTACAACCGATCCACCTCAC-3′ and 5′-ACTTTGCCTCCCAGATCACAG-3′), IL-1β (5′-AAACCTCTTCGAGGCACAAG-3′ and 5′-GTTTAGGGCCATCAGCTTCA-3′), IL-18 (5′-ACTGTACAACCGCAGTAATACGC-3′ and 5′-AGTGAACATTACAGATTTATCCC-3′), IL-22 (5′-TGAATAACTAACCCCCTTTCCCTG-3′ and 5′-TGGCTTCCCATCTTCCTTTTG-3′), IL-8 (5′-TAGCAAAATTGAGGCCAAGG-3′ and 5′-AAACCAAGGCACAGTGGAAC-3′), and IL-6 (5′-GGTACATCCTCGACGGCATCT-3′ and 5′-GTGCCTCTTTGCTGCTTTCAC-3′) were used for amplification in triplicate assays. On completion of polymerase chain reaction amplification, a deoxyribonucleic acid (DNA) melting curve analysis was carried out in order to confirm the presence of a single amplicon. β-Actin was used as an internal reference gene in order to normalize the transcript levels. Relative messenger RNA levels (2^−ΔΔCt^) were determined by comparing the polymerase chain reaction amplification cycle thresholds (Ct) for the gene of interest and β-actin gene (ΔCt) and ΔCt values for treated and control groups (ΔΔCt).

Given that APO, regardless BV presence, has been shown to be associated with cytokine levels, quantitative real-time polymerase chain reaction results of pregnant women that presented APO were interpreted separately from pregnant women without APO.

### Next-Generation Sequencing

Next-generation sequencing libraries were prepared by amplification of hypervariable regions of the 16S ribosomal DNA gene with the Ion 16S Metagenomics kit, followed by library generation using the Ion Plus Library kit (Life Technologies, Carlsbad, CA). Barcoded libraries were quantified and assessed for quality using the Agilent 2100 BioAnalyzer (Agilent Technologies). Libraries were pooled in equimolar amounts and sequenced on an Ion PGM Platform using a Ion 314 Chip Kit v2 and the Ion PGM Sequencing 400 kit (Life Technologies).

### Statistical Analysis

Statistical analysis was carried out using Prism 6 software (GraphPad). One-way analysis of variance (ANOVA) followed by multiple comparison tests or *t* test was used for all comparisons. Significance was accepted at *P* < 0.05.

## RESULTS

A total of 402 vaginal self-samples from pregnant women in their first trimester were screened by Nugent score. BV and IF were found in 28 and 21 pregnant women, respectively. Among the 28 pregnant women with BV, 5 miscarried between 18 and 22 completed weeks of amenorrhea. For the study, 4 groups were individualized post hoc: pregnant women with BV but without APO (BV-APO^−^; n = 23), pregnant women with BV and developing APO (BV-APO^+^; n = 5), pregnant women with IF (n = 21), and random samples from pregnant women with normal Nugent screening (controls; n = 28).

The general characteristics of the studied population are presented in Table 1. Antimicrobial treatment by clindamycin was noted for 5 pregnant women of our cohort in which 4 women belonged to BV-APO^+^ group and 1 to BV-APO^−^ group. Neutrophil count was under 20 cells per field at a 10-fold magnification for 26/28 BV, 19/21 IF, and 25/28 controls (data not shown). Finally, no history of sexually transmitted disease was reported in our cohort.

**TABLE 1:**
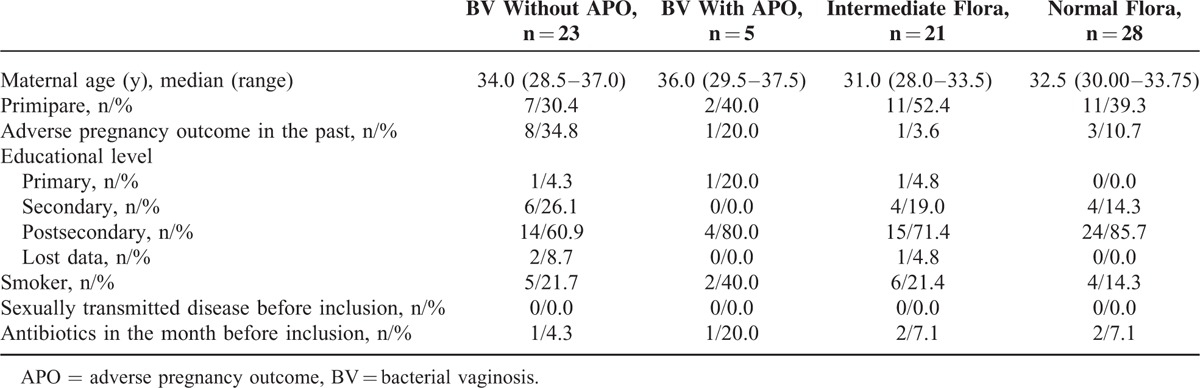
Population Characteristics of Studied Pregnant Women

**FIGURE 1:**
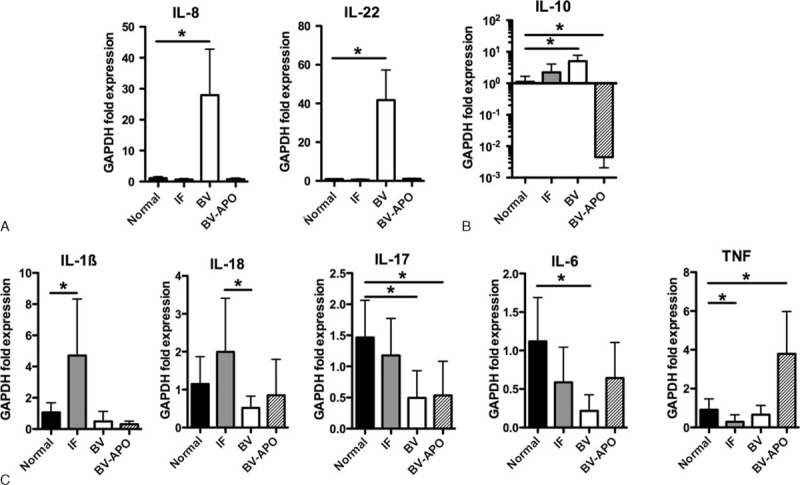
Relative gene expression of proinflammatory, mucosal homeostatic, and anti-inflammatory cytokines in the vagina pregnant women. APO = adverse pregnancy outcome, BV = bacterial vaginosis, IF = intermediate flora.

BV-APO^−^ pregnant women showed significantly increased IL-22, IL-10, and IL-8 expression in vaginal samples compared to controls (Figure 1A and B). Additionally, pregnant women with BV showed significantly decreased IL-17 and IL-6 expression (Figure 1C). IL-1β, IL-10, and TNF expression did not significantly differ (Figure 1C). For pregnant women with IF compared to controls, IL-1β was the only observed increase in cytokine expression.

Among the 5 BV-APO^+^ pregnant women, IL-10 expression decreased 100-fold in vaginal samples compared with controls and more than 1000-fold compared with BV-APO^−^ (Figure 1B). IL-8 and IL-22 messenger RNA were not significantly increased in these 5 samples compared with controls and differed significantly compared with BV-APO^−^ (Figure 1A). By contrast, TNF expression was increased compared with both controls and BV-APO^−^ (Figure 1C).

**FIGURE 2:**
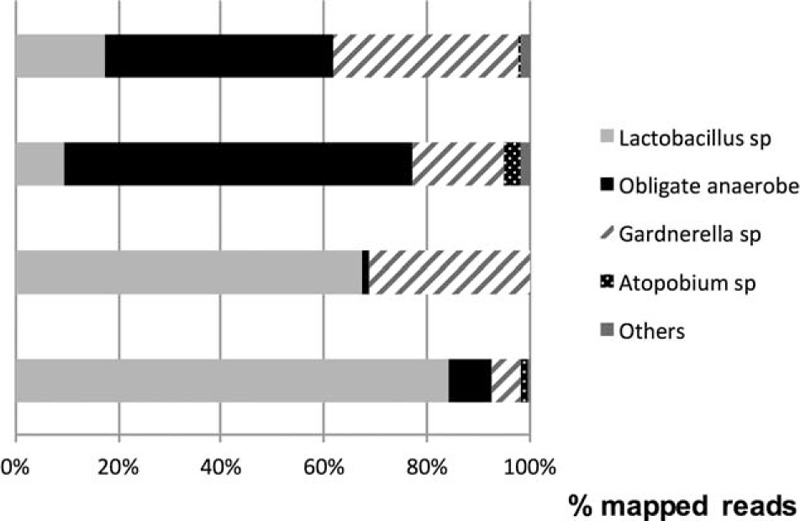
Vaginal microbiota composition of pregnant women with bacterial vaginosis but without adverse pregnancy outcome (BV-APO^−^) and pregnant women with bacterial vaginosis and developing adverse pregnancy outcome (BV-APO^+^).

Given these results, we wanted to find out if the vaginal microbiota composition might differ between BV-APO^+^ pregnant women and BV-APO^−^ pregnant women. Next-generation sequencing of 2 patients with BV-APO^+^ revealed that the vaginal microbiota was largely dominated by *Lactobacillus* sp, which represent 84.2% and 67.5% of the reads in the 2 patients with BV-APO^+^. *Lactobacillus iners* was the only *Lactobacillus* sp found (84.2% of the reads) for 1 patient and *L. iners* and *L. jensenii* were the only 2 *Lactobacillus* sp found for the second patient. Additionally, obligate anaerobes were drastically decreased in both BV-APO^+^ parturients when compared with BV-APO^−^ pregnant women (Figure 2). Indeed, the microbiota of these 2 BV-APO^−^ pregnant women were characterized by a high proportion of sequences from obligate anaerobe (67.5% and 44.4% in BV-APO^−^ compared with 8.3% and 1.4% in BV-APO^+^).

Finally given that *Lactobacillus* sp dominate the BV-APO^+^ pregnant women, we verified the Gram stain performed on the vaginal smear to establish the Nugent score. Gram-variable polymorphous bacilli and gram-variable rod-shaped bacilli characterized the smears but not the typical gram-positive bacilli with regular parallel edges morphotype of *Lactobacillus* sp.

## DISCUSSION

**FIGURE 3:**
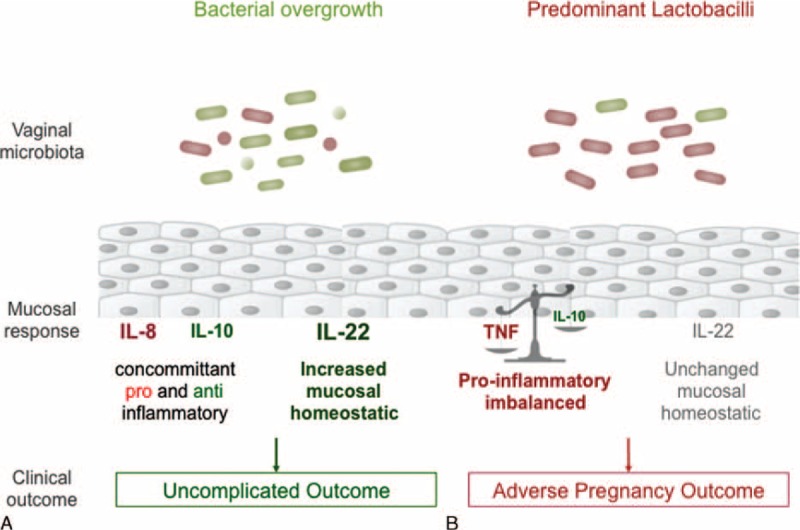
Proposed underlying mechanisms of outcomes of pregnancy in the setting of bacterial vaginosis. (A) The typical microbiota observed in bacterial vaginosis (varied obligate anaerobic bacteria and limited *Lactobacilli* and *Gardnerella*) elicit a mucosal response characterized by concomitant increases in both pro- and anti-inflammatory cytokines (IL-8 and IL-10, respectively) further balanced by an increased mucosal homeostatic response (increased IL-22) when compared to controls without bacterial vaginosis. This microbiota/response profile is associated with uncomplicated pregnancy outcomes despite bacterial vaginosis. (B) Another vaginal microbiota observed in certain cases of bacterial vaginosis (predominant *Lactobacilli* and lacking obligate anaerobes) elicit a different mucosal response characterized by a proinflammatory imbalance (increased TNF and 100-fold decrease in IL-10) and an unchanged homeostatic mucosal response (unchanged IL-22) when compared to controls without bacterial vaginosis. This microbiota/response profile is associated with adverse pregnancy outcomes. IL = interleukin, TNF = tumor necrosis factor.

Our results show that in pregnant women, BV without APO is associated with a vaginal immune response consisting in increased IL-22, IL-8, and IL-10 expression. In contrast, BV with APO is associated with decreased IL-10, unmodified IL-22/IL-8, and increased TNF expression compared with healthy controls (Figure 3). Although a limited pilot study, with the inherent risk of selection bias, our study was conducted in samples from a very general population of pregnant women and not a specific population at very high risk of BV. Of note, our population is exempt of patients with known histories of sexually transmitted diseases that could modify the vaginal immune response.

While high neutrophil recruitment is a strong marker of vaginitis due to sexually transmitted bacteria,^10^ we observe limited neutrophil recruitment in BV as previously reported.^11^ This observation is in contradiction with the fact that IL-8 is known to chemo-attract immune cells such as neutrophils to the site of injury.^12^ However, it is also well-known that during pregnancy an immune “re-balancing” toward IL-10 synthesis occurs to tolerate the fetus.^6,13^ We observe such an IL-10 increase in BV without APO. This IL-10 increase can explain the significant decrease we observe in other proinflammatory cytokines such as IL-6 and IL-17. In turn by inhibiting proinflammatory mediators effects at the materno-fetal interface,^14,15^ this increase in IL-10 could explain why concomitant IL-8-dependent neutrophil recruitment is limited. Additionally, IL-10 increase is consistent with an immune cellular profile we observed in an attempt at flow cytometry of vaginal samples of BV without APO (n = 3) which showed expansion of CD4^+^ T-lymphocytes and natural killer cells compared with controls from parturients without BV nor APO (n = 3) (data not shown). Indeed, IL-10 produced by both natural killer cells and subsets of CD4^+^ T-lymphocytes (T-regulatory lymphocytes) at the materno-fetal site^6,16,17^ is known to inhibit CD8^+^ T-lymphocyte expansion.

The most marked cytokine profiles are an increased vaginal anti-inflammatory response in BV with successful pregnancy outcome while this response is deeply depressed in BV with APO. Outside of the setting of BV, an anti-inflammatory state of the mother's immune system has been shown to be associated with successful pregnancy outcome.^6^ Conversely, increased expression of proinflammatory cytokines such as TNF is usually associated with several APOs.^18–21^ Thus, our finding that the vaginal anti-inflammatory immune response to BV we observe may participate in pregnancy outcome in this setting.

Ours is the first study to report that the response to BV with successful pregnancy outcome includes a major (40-fold) increase in IL-22 while this specific response is lacking in BV with APO. Upon damage, IL-22 participates in mucosal homeostasis by inducing mucosal antimicrobial peptide and mucus synthesis, by promoting wound repair and reinforcement of epithelial tight junctions, and by avoiding invasion by commensal bacteria.^8,22^ Potential for damage requiring IL-22-dependent mechanisms of protection exists in BV. Indeed, vaginal microbiota synthesizes several virulence factors such as sialidase, which can lyse mucins, favor bacterial adherence, and counteract immunoglobulin A protection leading in fine to mucosal damage.^23,24^ This novel finding suggests several possibilities. First, the combined IL-10 anti-inflammatory response with an increase in the IL-22 mucosal homeostatic response may be the protective appropriate vaginal immune response to BV. Second, the major known mucosal homeostatic roles of IL-22, antimicrobial peptide expression, mucin expression, tight junction reinforcement, and epithelial wound repair, may be enough in themselves to determine BV outcome.

Taken as a whole, the profile we observe in vaginal swabs of pregnant women with BV but without APO may consist in an adapted anti-inflammatory (5-fold IL-10 increase), strong mucosal homeostatic (40-fold IL-22 increase) response to BV-induced inflammation (28-fold IL-8 increase, with neutrophil recruitment limited by the concomitant anti-inflammatory response).

Interestingly, this cytokine profile is associated with a shift of the vaginal microbiota characterized by a dramatic decrease of obligate anaerobic bacteria and an increase of *L. iners* or *L. jensenii* that dominate the vaginal microbiota. Both *L. iners* and *L. jensenii* belong to the normal vaginal microbiota. While a high proportion of *L. iners* in the vaginal microbiota have been reported as associated with prematurity^25,26^ implying a pathogenic causative role. However, an increase in the amount of *L. iners* in the vaginal bacteria can be considered an indirect marker of a transitional change of the microbiota under environmental conditions such as those observed following antimicrobial treatment of BV.^27^ Furthermore, the previous studies showing the association between dominant *L. iners* group in the vaginal microbiota and prematurity did not study concomitant anti-inflammatory IL-10 and IL-22 synthesis. Indeed, in our cohort, neither *L. iners* nor *L. jensenii* are associated with IL-22 increase implying that these bacteria probably did not induce mucosal injury.

Obligate anaerobes, however, are known to synthesize strong inducers of mucosal IL-10 expression such as butyrate in the digestive tract.^28^ Therefore, the major decrease of anaerobes in the vaginal microbiota of BV-APO^+^ women could participate in the concomitant observed decrease in vaginal IL-10. Furthermore, oral and vaginal anti-anaerobic metronidazole treatment of BV has been shown to be associated with reduced levels of both IL-8 and IL-10.^29^ Taken together, our results may provide a possible explanation to the observed inefficiency of antimicrobials to prevent BV-related APO in clinical trials.^30^

Taken as a whole, our results highlight that “debugging” the vaginal microbiotal metabolome by determining the quantity of organic compounds synthesized by the microbiota known to induce anti-inflammatory mucosal responses or mucosal damage in different groups of pregnant women would be a relevant goal for further study. Finally, increasing the study sample size may allow confirmation of our results and possible identification of different cytokine/microbiota profiles.
